# Changes in the Active, Dead, and Dormant Microbial Community Structure across a Pleistocene Permafrost Chronosequence

**DOI:** 10.1128/AEM.02646-18

**Published:** 2019-03-22

**Authors:** Alexander Burkert, Thomas A. Douglas, Mark P. Waldrop, Rachel Mackelprang

**Affiliations:** a Department of Biology, California State University, Northridge, California, USA; b U.S. Army Cold Regions Research and Engineering Laboratory, Fort Wainwright, Alaska, USA; c Geology, Minerals, Energy, and Geophysics Science Center, U.S. Geological Survey, Menlo Park, California, USA; Kyoto University

**Keywords:** 16S rRNA, permafrost, microbial ecology

## Abstract

Permafrost hosts a community of microorganisms that survive and reproduce for millennia despite extreme environmental conditions, such as water stress, subzero temperatures, high salinity, and low nutrient availability. Many studies focused on permafrost microbial community composition use DNA-based methods, such as metagenomics and 16S rRNA gene sequencing. However, these methods do not distinguish among active, dead, and dormant cells. This is of particular concern in ancient permafrost, where constant subzero temperatures preserve DNA from dead organisms and dormancy may be a common survival strategy. To circumvent this, we applied (i) LIVE/DEAD differential staining coupled with microscopy, (ii) endospore enrichment, and (iii) selective depletion of DNA from dead cells to permafrost microbial communities across a Pleistocene permafrost chronosequence (19,000, 27,000, and 33,000 years old). Cell counts and analysis of 16S rRNA gene amplicons from live, dead, and dormant cells revealed how communities differ between these pools, how they are influenced by soil physicochemical properties, and whether they change over geologic time. We found evidence that cells capable of forming endospores are not necessarily dormant and that members of the class *Bacilli* were more likely to form endospores in response to long-term stressors associated with permafrost environmental conditions than members of the *Clostridia*, which were more likely to persist as vegetative cells in our older samples. We also found that removing exogenous “relic” DNA preserved within permafrost did not significantly alter microbial community composition. These results link the live, dead, and dormant microbial communities to physicochemical characteristics and provide insights into the survival of microbial communities in ancient permafrost.

**IMPORTANCE** Permafrost soils store more than half of Earth’s soil carbon despite covering ∼15% of the land area (C. Tarnocai et al., Global Biogeochem Cycles 23:GB2023, 2009, https://doi.org/10.1029/2008GB003327). This permafrost carbon is rapidly degraded following a thaw (E. A. G. Schuur et al., Nature 520:171–179, 2015, https://doi.org/10.1038/nature14338). Understanding microbial communities in permafrost will contribute to the knowledge base necessary to understand the rates and forms of permafrost C and N cycling postthaw. Permafrost is also an analog for frozen extraterrestrial environments, and evidence of viable organisms in ancient permafrost is of interest to those searching for potential life on distant worlds. If we can identify strategies microbial communities utilize to survive in permafrost, it may yield insights into how life (if it exists) survives in frozen environments outside of Earth. Our work is significant because it contributes to an understanding of how microbial life adapts and survives in the extreme environmental conditions in permafrost terrains.

## Abstract (precis)

Permafrost soils store more than half of Earth’s soil carbon despite covering ∼15% of the land area (C. Tarnocai et al., Global Biogeochem Cycles 23:GB2023, 2009, https://doi.org/10.1029/2008GB003327). This permafrost carbon is rapidly degraded following a thaw (E. A. G. Schuur et al., Nature 520:171–179, 2015, https://doi.org/10.1038/nature14338). Understanding microbial communities in permafrost will contribute to the knowledge base necessary to understand the rates and forms of permafrost C and N cycling postthaw. Permafrost is also an analog for frozen extraterrestrial environments, and evidence of viable organisms in ancient permafrost is of interest to those searching for potential life on distant worlds. If we can identify strategies microbial communities utilize to survive in permafrost, it may yield insights into how life (if it exists) survives in frozen environments outside of Earth. Our work is significant because it contributes to an understanding of how microbial life adapts and survives in the extreme environmental conditions in permafrost terrains.

## INTRODUCTION

Permafrost contains active microbial communities that are moderately diverse (1, 2). DNA-based methods, such as metagenomics and 16S rRNA gene sequencing, are commonly used to interrogate these communities with the underlying assumption that the data represent intact viable cells or that nonviable cells do not strongly affect conclusions drawn from whole-community DNA. However, DNA from dead cells may drastically alter estimates of diversity and abundance. Furthermore, because many communities host dormant cells, DNA-based approaches do not represent active members. In temperate soils, up to 40% of DNA is from dead or compromised cells (3). In permafrost, the amount of relic DNA may be even higher because frozen conditions preserve DNA from dead cells. In nonpermafrost environments, multiomic approaches provide functional information from RNA and protein, largely overcoming the problems of dormancy and relic DNA. In permafrost, this strategy has been successfully applied only in young near-surface permafrost due to low biomass and activity in older samples (4, 5). Therefore, in older, deeper permafrost, other methods must be used to differentiate between live, dead, and dormant cells.

Alternative methods to multiomics investigations, including microscopy, stable-isotope probing, and physiological measurements with microbial isolates, have been used on permafrost samples to demonstrate that an active community exists. Electron microscope examinations have shown evidence of apparently intact (no visible damage to cell envelopes), compromised (cell envelope ruptures), and dormant (endospores and cells with thick capsules) cells in permafrost (6–8). Using LIVE/DEAD differential staining coupled with fluorescence microscopy, Hansen et al. estimated that 26% of cells from a permafrost microbial community in Svalbard, Norway, were viable (2). Stable-isotope probing revealed that permafrost microorganisms can build biomass and replicate their genomes at subzero temperatures, as demonstrated through the incorporation of ^14^C-labeled acetate into lipids (9) and DNA (10). Similarly, studies involving permafrost microbial isolates have discovered microorganisms capable of reproduction at −15°C and metabolism down to −25°C (11, 12).

Though these studies show permafrost microbes exist in active states, dormancy is still a viable strategy for many taxa. Microorganisms enter dormancy in a variety of ways, though the hardiest and most persistent is the endospore formed by some Gram-positive taxa in response to nutrient limitation, temperature extremes, or other stressors (13). It is generally thought that endospores are able to survive for thousands to hundreds of thousands of years if embedded within a protective sample, such as an ice core (14) or permafrost (15, 16). However, endospore formation does not appear to be a universal survival strategy in permafrost, because the relative abundances of endospore-forming taxa vary substantially across the Arctic and sub-Arctic, ranging from vanishingly rare to almost 80% (16–18). The abundance may be related to soil physicochemical properties, including depth (19, 20), ice content, and permafrost age (18, 21). Furthermore, endospore-forming taxa in permafrost are not necessarily dormant. Hultman et al. used RNA-to-DNA ratios to show that *Firmicutes* in young Holocene permafrost are more active than expected based on DNA abundance alone, demonstrating that dormancy cannot be inferred based solely on 16S rRNA gene amplicon sequencing (4).

While endospore formation may contribute to long-term survival in permafrost, it is unclear whether this strategy is optimal across geologic timescales. Despite resistance to extreme conditions, DNA within an endospore can still accumulate damage (22). Typically, DNA damage is repaired upon germination by DNA repair machinery (13). However, damage accumulated over geologic timescales may be beyond the ability of repair enzymes to remedy. Willerslev et al. amplified 16S rRNA genes from globally distributed permafrost soils ranging from 0 to 600,000 years old and found non-endospore-forming *Actinobacteria* were more highly represented than the endospore-forming *Firmicutes* in samples of increasing age (>100,000 years) (21). Johnson et al. used a uracil-*N*-glycosylase treatment to break down damaged DNA extracted from ancient permafrost and found *Actinobacteria* rather than endospore-forming *Firmicutes* were more highly represented in the oldest samples (>600,000 years) (23). This suggests that metabolic activity and active DNA repair may be a better survival strategy than dormancy in increasingly ancient permafrost. Therefore, endospore formation may not be an optimal survival strategy in permafrost for timescales beyond the late Pleistocene.

**FIG 1:**
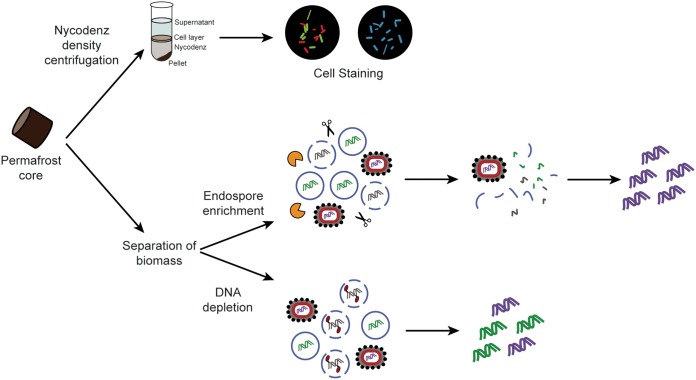
Experimental strategy overview. Live, dead, and dormant cell counts were conducted by separating cells from soil using Nycodenz density centrifugation, staining with a LIVE/DEAD differential stain or DAPI, and counting via fluorescence microscopy. For the endospore enrichment and dead cell depletion experiments, we separated biomass from soil using a gravity separation technique. To deplete DNA from dead organisms, cell mixtures were treated with propidium monoazide and then exposed to light, causing cross-links with DNA not enclosed by an intact cell envelope or spore coat. The cross-links inhibit downstream PCR amplification. To enrich for endospores, cell mixtures were exposed to lysozyme, heat, and DNase, which lyses vegetative cells and degrades DNA. In both the endospore enrichment and dead cell depletion experiments, the 16S rRNA gene was amplified and used for downstream analysis.

**FIG 2:**
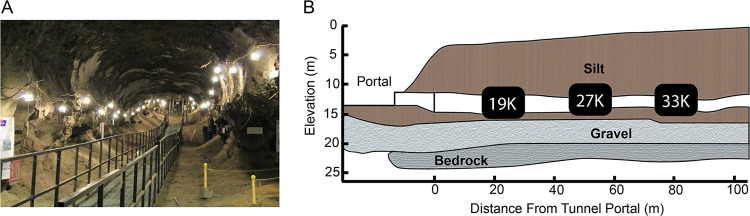
(A) Photograph taken by A. Burkert from the portal to the CRREL Permafrost Tunnel. (B) Schematic drawing of the northern section of the CRREL Permafrost Tunnel identifying where our samples were collected. The age of exposed permafrost increases with distance inward from the tunnel portal. Figure adapted from Mackelprang et al. (18), and panel B redrawn from Bjella et al. (80) and Hamilton et al. (24).

Here, we combine three strategies to address the unresolved question of whether indicators of life (e.g., DNA, cells, and endospores) are from live, viable microbial communities or, instead, originate from only dead and dormant cells (Fig. 1). We hypothesized that dormancy would increase with age but endospore formation would not be the sole mechanism for survival. Viable non-endospore-forming taxa should also be present, and some endospore-forming cells should exist in a nondormant state. We also hypothesized that cell abundance would decrease with age but could also be affected by the physiochemical conditions of the permafrost (Fig. 2). Further, we asked whether preserved DNA from dead cells is so compositionally dissimilar from DNA from live cells that it alters inferences about microbial community structure in permafrost made with total soil DNA.

## RESULTS

### Soil chemistry.

Soil physicochemical properties, including ice content, carbon, nitrogen, pH, and conductivity, varied significantly among age categories (Kruskal-Wallis; *P* < 0.01 for all measurements) (Table 1). These values were consistently higher in the intermediate age category than in the older and younger samples.

**TABLE 1:** Permafrost physicochemical characteristics across the three time periods

Parameter	Value^*a*^							
	19,000 yr	*P* value (19,000 yr vs 27,000 yr)	27,000 yr	*P* value (27,000 yr vs 33,000 yr)	33,000 yr	*P* value (33,000 yr vs 19,000 yr)	Kruskal-Wallis	
							χ^2^ (df = 2)	*P* value
Ice content (%)	27.70 ± 1.73	**0.001**	50.01 ± 4.16	0.077	35.30 ± 0.50	0.077	12.5	**0.002**
Total C (%)	1.64 ± 0.18	**0.003**	3.47 ± 0.19	0.229	3.06 ± 0.08	0.061	10.82	**0.004**
Organic C (%)	1.62 ± 0.16	**0.014**	2.99 ± 0.30	0.724	2.76 ± 0.06	**0.020**	9.5	**0.009**
Total N (%)	0.16 ± 0.02	**0.013**	0.30 ± 0.01	0.943	0.29 ± 0.01	**0.013**	9.45	**0.009**
C/N ratio	10.45 ± 0.34	**0.013**	11.70 ± 0.11	0.944	10.62 ± 0.17	**0.013**	9.38	**0.009**
pH	7.32 ± 0.04	0.203	7.46 ± 0.05	**0.004**	6.88 ± 0.10	0.084	10.26	**0.006**
EC (dS/m)	0.39 ± 0.02	**0.003**	0.87 ± 0.04	0.072	0.46 ± 0.03	0.179	11.18	**0.004**
DOC (ppm)	3,141 ± 162	**0.009**	9,338 ± 1,649	0.524	5,776 ± 212	**0.029**	9.78	**0.008**

a The values are averages of five replicates ± 1 standard error of the mean. Statistical differences were tested using a Kruskal-Wallis test and Dunn’s *post hoc* test, with *P* values corrected using the false-discovery rate. Significant *P* values (*P* < 0.05) are shown in boldface.

### Cell enumeration.

**FIG 3:**
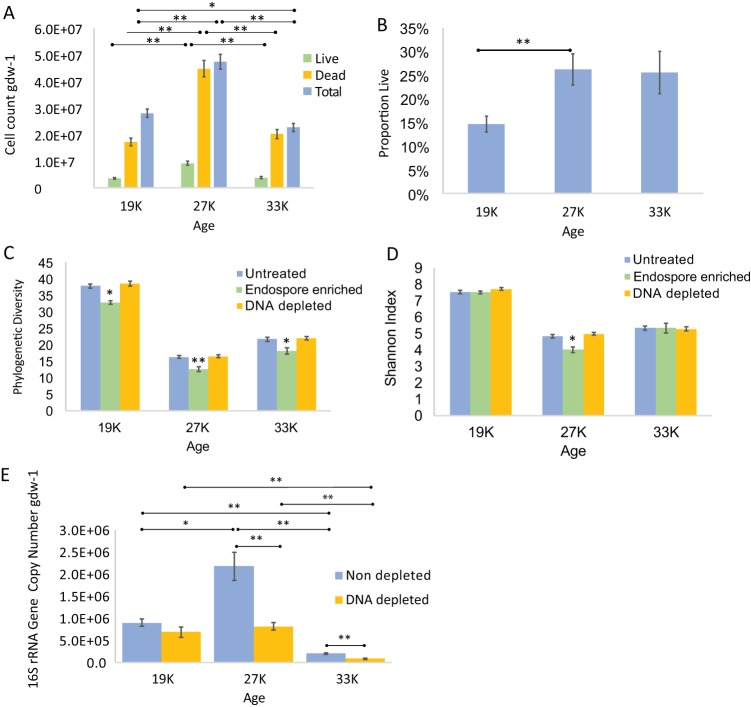
(A) Direct cell counts as determined by cell staining and fluorescence microscopy. Shown are live, dead, and total (DAPI) counts. (B) Proportion of live cells as determined by direct counts of live cells (stained with SYTO 9) and total cells (stained with DAPI). (C) Phylogenetic diversity index compared across age categories and treatment types. (D) Shannon index compared across age categories and treatment types. (E) 16S rRNA gene copy numbers in samples depleted of dead DNA using propidium monoazide and nondepleted controls. qPCR of the V4 region of the 16S rRNA gene in samples showed that average copy numbers decreased for all age groups in the propidium monoazide-treated group (DNA-depleted samples) compared to the untreated group (nondepleted controls). The values show the averages of five replicate cores, and the error bars show the standard errors of the mean. Significant *P* values as tested by Dunn’s *post hoc* test are indicated (*, *P* < 0.05; **, *P* < 0.01).

We performed cell counts across the chronosequence using LIVE/DEAD and DAPI (4′,6-diamidino-2-phenylindole) staining coupled with fluorescence microscopy. Average cell counts ranged from 3.6 × 10^6^ to 9.2 × 10^6^ cells g (dry weight)^−1^ (live cells), 1.7 × 10^7^ to 4.5 × 10^7^ cells g (dry weight)^−1^ (dead cells), and 2.3 × 10^7^ to 4.7 × 10^7^ cells g (dry weight)^−1^ (total cell count). Live (Kruskal-Wallis; χ^2^ = 46.25 [df = 2]; *P* < 0.001), dead (Kruskal-Wallis; χ^2^ = 53.16 [df = 2]; *P* < 0.001), and total (Kruskal-Wallis; χ^2^ = 53.58 [df = 2]; *P* < 0.001) counts were significantly different among categories and higher in the intermediate-age samples than in the oldest (Dunn’s test; *P* < 0.01) and youngest (Dunn’s test; *P* < 0.01) samples (Fig. 3A). Counts for DAPI were higher than counts based on live plus dead cells for the youngest samples (2.80 × 10^7^ ± 1.57 × 10^6^). However, for the intermediate-age (4.47 × 10^7^ ± 2.80 × 10^6^) and oldest (2.27 × 10^7^ ± 1.46 × 10^6^) samples, counts for DAPI were lower than counts based on live plus dead cells.

The proportion of live cells was significantly higher in intermediate-age samples (26%) than in the youngest samples (14%) (Kruskal-Wallis, χ^2^ = 9.84 [df = 2]; *P* < 0.01; Dunn’s test, *P* < 0.01) (Fig. 3B). In the oldest samples, 25% of the cells were alive, though this was not significantly different than the values observed for the youngest or intermediate-age samples. The ratio of live cells to dead cells did not change significantly across the chronosequence (Kruskal-Wallis; χ^2^ = 1.38; *P* > 0.05).

### Depletion of DNA from dead cells via propidium monoazide treatment.

To determine if DNA from dead cells biases estimates of taxonomic relative abundance from the whole community, we used a propidium monoazide (PMA) treatment to deplete dead-cell DNA. Depletion did not significantly change the relative abundances of taxa at the phylum, class, order, or family level for any of the age categories (Mann-Whitney-Wilcoxon test; *P* > 0.05). *Actinobacteria* were consistently less abundant in depleted samples than in nondepleted controls. Propidium monoazide treatment did not change alpha diversity measurements—there were no significant differences in depleted samples compared with nondepleted controls (Kruskal-Wallis; *P* > 0.05) (Fig. 3C and D).

To confirm that propidium monoazide treatment successfully depleted DNA from dead and membrane-compromised cells, we determined 16S rRNA gene copy numbers in treated and untreated samples using quantitative PCR (qPCR) (*R*^2^ = 0.99; 45.5% efficiency). Depletion decreased copy numbers for the youngest (∼24%; Student's *t* test; *P* > 0.05), intermediate-age (∼62%; Student's *t* test; *P* < 0.01), and oldest (∼58%; Student's *t* test; *P* < 0.01) samples (Fig. 3E). 16S rRNA gene copy numbers varied significantly across ages for the depleted samples (Kruskal-Wallis; χ^2^ = 25.05 [df = 2]; *P* < 0.001) and control samples (Kruskal-Wallis; χ^2^ = 31.25 [df = 2]; *P* < 0.001). The oldest samples had significantly fewer 16S rRNA gene copies than the youngest (Dunn’s test; *P* < 0.01) and intermediate-age (Dunn’s test; *P* < 0.01) samples for both the depleted and control samples (Fig. 3E).

### Endospore enrichment.

**FIG 4:**
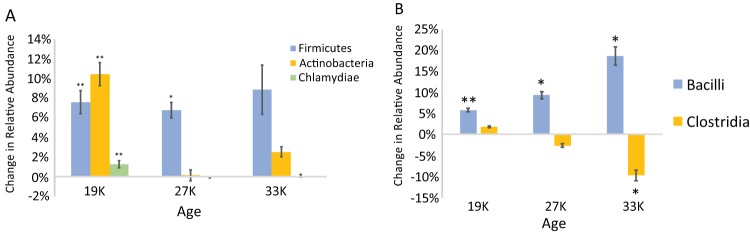
(A) Phylum level changes in relative abundance due to endospore enrichment. (B) Class level changes in relative abundance due to endospore enrichment. The values show averages of five replicate cores, and the error bars show standard errors of the mean. Significant *P* values as tested by the Mann-Whitney-Wilcoxon test are indicated (*, *P* < 0.05; **, *P* < 0.01).

The community of cells remaining after endospore enrichment had reduced alpha diversity compared with nontreated controls as measured via phylogenetic richness (Kruskal-Wallis; *P* < 0.05) (Fig. 3C) and the Shannon index (Kruskal-Wallis; *P* < 0.05) (Fig. 3D). The enzyme treatment increased the relative abundances of three phyla—*Firmicutes*, *Actinobacteria*, and *Chlamydiae* (Fig. 4A). Endospore enrichment tended to increase the relative abundance of *Firmicutes* in all age categories but was significant only for the youngest (Mann-Whitney-Wilcoxon test; *U* = 0; *P* < 0.01) and intermediate (Mann-Whitney-Wilcoxon test; *U* = 2; *P* < 0.05) age categories. At the class level, endospore enrichment changed the abundances of *Bacilli* and *Clostridia*, but in opposing directions. It increased the relative abundance of *Bacilli* across all age categories, growing more pronounced in the older samples (youngest, 5.8%; intermediate, 9.3%; oldest, 18.6%). These data were significant for each age category (Mann-Whitney-Wilcoxon test; *U* = 2; *P* < 0.05) (Fig. 4B). In contrast, endospore enrichment significantly decreased the relative abundance of *Clostridia* in the oldest age category (10%) (Mann-Whitney-Wilcoxon test; *U* = 2; *P* < 0.05) (Fig. 4B). Treatment did not significantly change the relative abundance of *Clostridia* in the youngest and intermediate-age samples. These trends were driven by the families *Planococcaceae*, *Thermoactinomycetaceae*, *Bacillaceae*, and *Paenibacillaceae* for *Bacilli* and the family *Clostridiaceae* for *Clostridia* (Table 2).

**TABLE 2:** Average percent difference in relative abundance between endospore-enriched samples and nonenriched controls across the three age categories

Taxon	Family	Difference^*a*^ (%)					
		19,000 yr	*U* value	27,000 yr	*U* value	33,000 yr	*U* value
*Actinobacteria*	*Micrococcaceae*	2.4 ± 0.5^*b*^	0	0.0 ± 0.0	9.5	0.0 ± 0.0	11
	*Solirubrobacteraceae*	1.5 ± 0.4	3	0.6 ± 0.3	4	0.1 ± 0.1	5
	*Gaiellaceae*	1.0 ± 0.3^*c*^	1	−0.2 ± 0.1	8	0.1 ± 0.1	8.5
*Bacilli*	*Planococcaceae*	2.4 ± 0.9	4	−8.5 ± 1.0^*c*^	1	4.0 ± 2.6	8
	*Thermoactinomycetaceae*	0.4 ± 0.1^*c*^	0	20 ± 2.5^*c*^	2	7.8 ± 2.2	6
	*Bacillaceae*	0.7 ± 0.1^*c*^	0	−2.5 ± 1.2	8	1.0 ± 0.4	9
	*Paenibacillaceae*	2.5 ± 0.4^*c*^	0	0.4 ± 1.6	12	5.6 ± 2.7	4
*Clostridia*	*Clostridiaceae*	3.1 ± 0.4^*b*^	0	−2.5 ± 1.5	5	−9.2 ± 1.2^*b*^	0

a A negative value shows underrepresentation in the endospore-enriched samples compared to the nonenriched controls, while a positive value shows overrepresentation. The values are averages of five replicate cores. The *U* value is a test statistic generated by a Mann-Whitney-Wilcoxon test that represents the level of difference between two groups. Lower *U* values indicate that two groups are more different.

b *P* < 0.01 (Mann-Whitney-Wilcoxon test).

c *P* < 0.05 (Mann-Whitney-Wilcoxon test).

Endospore enrichment increased the relative abundance of *Actinobacteria* in the youngest age category from 22.4% to 32.8% (Mann-Whitney-Wilcoxon test; *U* = 0; *P* < 0.01) (Fig. 4A). This increase was driven by the families *Micrococcaceae* within the *Actinomycetales*, the *Gaiellaceae*, and *Solirubrobacteraceae* (Table 2). There were no significant differences in *Actinobacteria* relative abundance due to endospore enrichment in the intermediate-age and oldest samples.

*Chlamydiae* relative abundance increased in the youngest age category from 0.7% to 2.0% as a result of endospore enrichment (Mann-Whitney-Wilcoxon test; *U* = 0; *P* < 0.01) (Fig. 4A). This trend was driven by an increase in the relative abundance of the family *Chlamydiaceae*. All other major taxa, including *Proteobacteria*, *Alphaproteobacteria*, *Deltaproteobacteria*, *Bacteroidetes*, *Acidobacteria*, *Chloroflexi*, and *Planctomycetes*, showed significant decreases in relative abundance due to the endospore enrichment (see Table S2 in the supplemental material).

### 16S rRNA gene-based community analysis.

**FIG 5:**
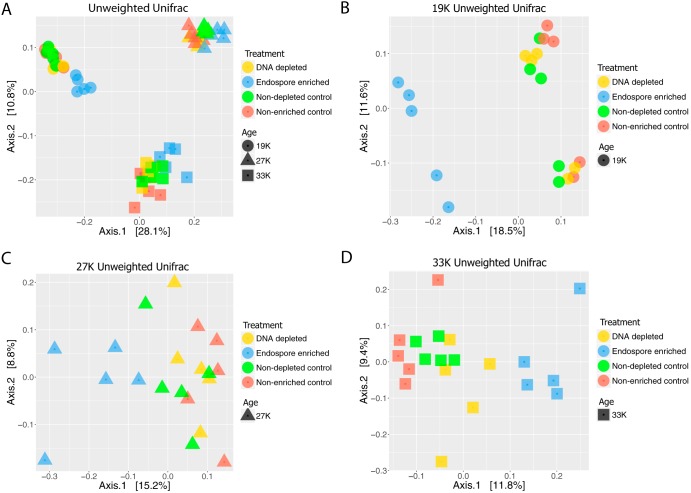
PCoA based on weighted UniFrac distances. (A) PCoA plots of all ages and treatments. (B to D) PCoA plots by age. Depleted, samples that underwent depletion of dead DNA; nondepleted controls, samples that were extracted together but did not receive the propidium monoazide treatment; endospore enriched, samples that underwent endospore enrichment; nonenriched controls, samples that were extracted in parallel but did not undergo treatment. Samples differed significantly by age (PERMANOVA; *R*^2^ = 0.83, *F* = 135.64, *P* < 0.001) (A) and treatment for the youngest (PERMANOVA; *R*^2^ = 0.51, *F* = 5.65, *P* < 0.001) (B), the intermediate (PERMANOVA; *R*^2^ = 0.42, *F* = 3.87, *P* < 0.01) (C), and the oldest (PERMANOVA; *R*^2^ = 0.37, *F* = 3.14, *P* < 0.01) (D) samples.

To explore how experimental treatments influenced overall community structure, we compared 16S rRNA gene sequences from 19,000-, 27,000-, and 33,000-year-old permafrost from treated and control samples (*n* = 5 replicate samples for 3 age categories for 4 treatments, or 60 samples total). Comparisons of diversity between samples revealed that samples clustered by age regardless of treatment (Fig. 5A). When comparing the samples within each age category, endospore-enriched samples consistently clustered separately from controls and DNA-depleted samples (Fig. 5B to D). Phyla that differed significantly based on age and treatment (endospore enrichment and depletion of DNA from dead cells) using a linear mixed effect model included *Proteobacteria*, *Actinobacteria*, *Firmicutes*, *Bacteroidetes*, *Chloroflexi*, *Acidobacteria*, *Planctomycetes*, and *Chlamydiae* (false-discovery rate [FDR]-corrected *P* < 0.01).

Overall, alpha diversity estimates were highest in the youngest category and lowest in the intermediate-age category (Table 3). All soil chemistry measurements correlated significantly with nonmetric multidimensional scaling vectors (permutational multivariate analysis of variance [PERMANOVA]; *P* < 0.05) (see Fig. S2 in the supplemental material). Age and ice content were the strongest drivers of community structure (PERMANOVA, *R*^2^ = 0.49, *F* = 181.35, *P* < 0.001; PERMANOVA, *R*^2^ = 0.26, *F* = 91.98, *P* < 0.001). Other measurements had a small but significant effect on community structure: dissolved organic carbon (DOC) (PERMANOVA; *R*^2^ = 0.04; *F* = 13.13; *P* < 0.001), C/N ratio (PERMANOVA; *R*^2^ = 0.03; *F* = 12.27; *P* < 0.001), total C (PERMANOVA; *R*^2^ = 0.02; *F* = 6.63; *P* < 0.01), and total N (PERMANOVA; *R*^2^ = 0.01; *F* = 3.43; *P* < 0.05).

**TABLE 3:** Alpha diversity estimates

Sample	Age (yr)	Alpha diversity^*a*^					
		Shannon index			PD		
		Avg	SE	*P* value	Avg	SE	*P* value
Untreated	19,000	7.53	0.10	**1.3 × 10^−5^**^*b*^	37.82	0.69	**1.7 × 10^−6^**^*b*^
	27,000	4.84	0.11	0.1150^*c*^	16.25	0.43	**1.7 × 10^−2^**^*c*^
	33,000	5.33	0.12	**3.8 × 10^−3^**^*d*^	21.63	0.58	**1.3 × 10^−2^**^*d*^
DNA depleted	19,000	7.70	0.09	**3.4 × 10^−3^**^*b*^	38.51	0.71	**1.2 × 10^−3^**^*b*^
	27,000	4.98	0.08	0.2293^*c*^	16.50	0.34	0.0771^*c*^
	33,000	5.28	0.13	0.0605^*d*^	21.92	0.46	0.0771^*d*^
Endospore enriched	19,000	7.50	0.08	**1.6 × 10^−3^**^*b*^	32.83	0.53	**1.6 × 10^−3^**^*b*^
	27,000	4.00	0.18	0.1039^*c*^	12.55	0.79	0.1039^*c*^
	33,000	5.34	0.30	0.0990^*d*^	18.15	0.94	0.0990^*d*^

a Alpha diversity estimates based on 16S rRNA gene sequences for the untreated samples, PMA-treated samples, and endospore-enriched samples across the three age groups were measured using QIIME. Overall diversity was lowest in the intermediate-age samples. Within treatment types, differences between age groups were tested using a Kruskal-Wallis test and Dunn’s *post hoc* test. The values are averages of five replicates, and significant *P* values are shown in boldface.

b Versus 27,000 years.

c Versus 33,000 years.

d Versus 19,000 years.

## DISCUSSION

In this study, we used three complimentary methods—two of which have never been applied to permafrost—to distinguish between live, dead, and dormant cell types: LIVE/DEAD staining, endospore enrichment, and depletion of DNA from dead cells. We present evidence that the propensity to exist as an endospore (among endospore-forming taxa) was taxon dependent and that microbial cell counts and diversity were largely controlled by soil chemistry. These trends were driven by soil physicochemistry and/or showed a relationship to increasing permafrost age. We also found that removal of preserved DNA from dead organisms significantly altered microbial community composition. This study builds on previous work aimed at understanding how microbial communities adapt to the extreme conditions of permafrost over geologic timescales (18).

Under the stressful conditions associated with long-term interment in permafrost, members of the phylum *Firmicutes* became more abundant with increasing permafrost age (as shown in this study and a previous study from the Cold Regions Research and Engineering Laboratory [CRREL] tunnel [18]). One interpretation is that most are endospores, which enables their persistence while non-endospore formers succumb to harsh conditions. However, we show that many endospore formers exist as vegetative cells, but the tendency to do so differed among classes. Members of class *Bacilli* were more likely to exist as endospores in increasingly ancient permafrost, while members of *Clostridia* were more likely to remain in a nondormant state. This trend grew more pronounced as sample age increased. For *Bacilli*, this trend was driven by the families *Planococcaceae*, *Thermoactinomycetaceae*, *Bacillaceae*, and *Paenibacillaceae*, which are predominantly aerobes (25). For *Clostridia*, the trend was driven by the family *Clostridiaceae*, which decreased by over 9% in the endospore-enriched samples compared to nonenriched controls from the oldest category. Most members of the family *Clostridiaceae* are obligate anaerobes that depend primarily on substrate level phosphorylation for ATP generation (25). Permafrost is characterized by low reduction potential, indicative of limited oxygen availability (26), which could contribute to endospore formation for groups of *Bacilli* while anaerobic *Clostridia* are able to persist in a nondormant state. Taken together with the observation that *Bacilli* increased in relative abundance in the intermediate-age (76%) and the oldest (52%) samples compared to the youngest samples (3%) for the untreated controls, these data suggest endospore formation is a viable survival strategy for *Bacilli* against the conditions of ancient permafrost, at least for the timescales observed in this study. However, over increasing timescales, endospores can accumulate DNA damage. In the absence of active repair machinery, this damage may become lethal (13, 21, 23). Further investigations into even older permafrost may show decreases in endospore-forming taxa in favor of organisms that can actively repair DNA damage, as has been suggested previously (21).

The presence of endospore-forming *Firmicutes* is highly variable across Arctic permafrost, though they have been shown to increase in relative abundance over geologic timescales (18). Tuorto et al. (10) used stable-isotope probing to identify active community members and found that *Firmicutes* were not among those replicating their genomes at subzero temperatures, with the caveat that stable-isotope probing identifies only taxa that actively replicate their genomes. The study would not have identified cells that were metabolically active but not dividing during the experimental time frame (10). Other studies have demonstrated that endospore-forming taxa are active. For example, Hultman et al. found that the abundance of transcripts from *Firmicutes* exceeded expected levels based on the abundance of DNA sequence reads (4).

Though our endospore treatment was designed to enrich for true endospores, it also enriched for two other phyla—*Actinobacteria* and *Chlamydiae*—but only in the youngest age category. *Actinobacteria* were previously found to resist this treatment, perhaps due to their ability to form dormant and spore-like structures (27). In our samples, members of the families *Micrococcaceae*, *Solirubrobacteraceae*, and *Gaiellaceae* were overrepresented in our endospore-enriched group compared to nonenriched controls. Members of these families can survive radiation, starvation, and extreme desiccation (23, 25), which may also confer the ability to resist endospore enrichment.

Unexpectedly, *Chlamydiae* were also more abundant in the endospore-enriched samples than in nonenriched controls from the youngest age category. This was driven exclusively by the family *Chlamydiaceae*, whose members are not known to have resting states (25). All genera within this class are obligate intracellular symbionts of members of the genus *Acanthamoeba. Acanthamoeba* is a genus of single-celled eukaryotes commonly found in freshwater and soil. They exist in free-living forms and as stress-resistant dormant cysts (28), which could account for the increase in the relative abundance of *Chlamydiae* in the endospore-enriched samples. Several studies have found intact and viable *Acanthamoeba* cysts in Holocene and Pleistocene permafrost (29, 30). Though we did not sequence eukaryotic marker genes, it is possible that there are acanthamoebas in our samples. Alternatively, it could be that there are unknown resting mechanisms in members of the class *Chlamydiaceae*.

Depleting DNA from dead cells did not significantly alter microbial community composition, suggesting that sequencing 16S rRNA marker genes from total DNA provides a reasonable representation of community structure in these permafrost samples. This is expected when the death rate and the rate of degradation of dead cells are proportional among taxa (31). Similar findings have been observed for marine sediments (32). We note that this result may not be consistent across all permafrost. Studies in nonpermafrost soils from the continental United States suggest that physicochemistry influences whether relic DNA impacts community structure (3). Though depletion experiments did not bias estimates of relative abundance, they reduced 16S rRNA gene copy numbers by ∼48%, suggesting that ∼1/2 of the DNA from permafrost is relic DNA. Our estimate is higher than values taken from temperate soil, where ∼40% of DNA is relic (3).

One potential limitation of using the propidium monoazide treatment to deplete DNA from dead cells is that soil particles can prevent light penetration and limit efficacy. We attempted to mitigate this concern by extracting biomass from soil particles, performing intermittent mixing during treatment, and increasing the PMA concentration for particle-rich samples (33). We also confirmed the efficacy of the experiment by spiking samples with dead Escherichia coli cells and showed that even in the presence of soil particles, treatment removed 66% of added exogenous DNA.

Direct cell counts and analysis of 16S rRNA amplicons from permafrost soils showed that ice content drove cell abundance and microbial diversity, similar to a prior study conducted in the permafrost tunnel with samples collected from the same locations along the tunnel wall (18). While endospore enrichment significantly altered microbial community structure within each age group compared to nonenriched samples, the effects of age and ice content were still much stronger predictors of beta diversity among samples of all ages.

Microbial cell counts ranged from 2.3 × 10^7^ to 4.7 × 10^7^ cells g (dry weight)^−1^ and were consistent with previous studies from Arctic and sub-Arctic permafrost (1, 16, 17, 26, 34) but were 1 to 2 orders of magnitude lower than is commonly observed in temperate soils (35–37). Among the three age categories, the intermediate-age category had the highest numbers of live, dead, and total cells and the highest proportion of live cells. Similarly, 16S rRNA gene copy numbers from total community DNA and from samples depleted of dead DNA using propidium monoazide showed the highest counts in the intermediate-age category and the lowest counts in the oldest category. We note that direct counts of live cells did not match 16S rRNA gene copy numbers of DNA-depleted samples, though theoretically they should be roughly similar. This is likely because DNA extraction and cleanup kits substantially reduce DNA yields (38, 39). While we took every effort to maximize yields during extractions, this may have contributed to the differences observed between the two enumerations.

Though cell counts were highest in the intermediate-age category, alpha diversity was lower than in the youngest and oldest samples. The decrease in alpha diversity may reflect the high ice content in the cores, which has been shown previously to decrease diversity in permafrost (40, 41). The observation that cell counts were greatest in the intermediate-age samples is consistent with a previous publication from the permafrost tunnel chronosequence, though counts reported here are greater by an order of magnitude. This is likely due to increased cell recovery as a result of our biomass extraction protocol, which used sonication (rather than vortexing) to separate cells from soil particles (18).

The LIVE/DEAD staining approach uses membrane permeability as a proxy for viability and has been used extensively in environmental samples (43–45), including permafrost (2, 18). One potential drawback to using this approach is that live cells can incorrectly stain as dead, particularly under dark anoxic conditions (46). We suggest this is unlikely to impact our samples because permafrost is a generally stable environment in which membrane potentials are well maintained (47) and we stained under aerobic conditions in the light. However, if our treatment affected membrane potentials, it would result in an underestimate of the number of live cells.

Our initial hypothesis that age would drive patterns of dormancy, diversity, and live/dead cell abundances was partially supported. We found the anticipated chronological gradient for *Bacilli* and *Clostridia* relative abundances, but not for cell counts and diversity. Instead, they were controlled primarily by moisture and soil chemistry. We partially based our age-related hypotheses on data we previously generated from the CRREL tunnel, which showed a strong relationship between the abundance of survival-related genes and permafrost age (18). Taken together, the data from this and our prior study suggest a complex relationship between age, chemistry, and moisture. Expanding investigations into older permafrost samples and permafrost representing different biogeochemical properties will be essential to building a model for how microorganisms in permafrost survive. The question of dormancy is a key building block of this model. While dormancy appears to be a survival strategy for microbes not well adapted to life in permafrost (e.g., *Bacilli*, which are commonly aerobes or facultative anaerobes), those that appear to be better adapted (e.g., *Clostridia*, which are typically anaerobes and have a diverse suite of metabolic strategies to draw upon) are less likely to resort to dormancy. This suggests an increase in endospore formation among maladapted taxa upon entrance into the late Pleistocene but that metabolic activity may be increasingly necessary in older permafrost. Expanding the age range of permafrost and conducting further in-depth studies of current samples (such as quantitative measurements of endospore markers compared with vegetative-cell markers) will be crucial. If life exists on cryogenic bodies in the solar system, it must have persisted over longer timescales than exist in Earth’s permafrost. Thus, a chronosequence-based approach to understanding survival may allow us to extrapolate to more ancient cryoenvironments.

Finally, permafrost communities may have an important role in the biogeochemical cycling of elements and greenhouse gas production following thawing. Our data suggest DNA-based studies provide a reasonable representation of the taxonomic reservoir present and poised to decompose soil carbon upon thawing. They also demonstrate that the taxonomic diversity and the number of cells are governed by soil physicochemical characteristics of permafrost soil. Communities with less diversity and fewer cells may be slower to respond to thawing, which has implications for the speed at which carbon is processed and released into the atmosphere. Our data suggest that the reservoir (both the number of cells and the diversity) of microbes is controlled by carbon and moisture content and may be influenced by age and highlight the need for a detailed understanding of how physicochemical properties shape microbial communities across permafrost environments.

## MATERIALS AND METHODS

### Permafrost sample collection.

We collected frozen permafrost samples from the U.S. CRREL Permafrost Tunnel research facility located 16 km north of Fairbanks, Alaska (64.951^o^N, −147.621^o^W) (Fig. 2A). The facility includes 300 m of tunnels excavated into the permafrost. The tunnel where samples were collected extends 110 m horizontally at a depth of ∼15 m into a hillside, exposing a chronosequence of late Pleistocene permafrost (Fig. 2B) (24, 48). The temperature of the tunnel is maintained by refrigeration at −3°C. It contains massive ice wedges surrounded by high-organic-content ice-cemented windblown silt (49). In April 2016, we collected ice-cemented silt from three locations inside the tunnel representing three age categories: 19,000 (approximately 10 m from the portal), 27,000 (54 m), and 33,000 (88 m) years old, as determined previously by radiocarbon dating (18). After removing the sublimated surface layer (∼5 cm) from the walls of the tunnel, we collected five replicate cores per age category using a 7.5- by 5-cm keyhole saw attached to a power drill, as described previously (18). The cores were shipped back to California State University, Northridge (CSUN), on dry ice and stored at −20°C.

### Permafrost subsampling.

For subsampling, we placed cores on autoclaved foil at room temperature for 10 min to allow the outer ∼1 cm to thaw and soften. Surface contamination was removed by scraping off the outer layer with an autoclaved knife to expose the uncontaminated frozen interior. We subsectioned the remaining uncontaminated material, using a fresh knife, into sterile 50-ml Falcon tubes and high-density polyethylene bags in preparation for downstream treatment.

### Soil chemistry.

Ice content was measured via gravimetric moisture analysis. To determine pH, soil was diluted 1:1 in a 0.01 M CaCl_2_ solution and measured using a Hanna benchtop meter with attached probe (Hanna Instruments, Woonsocket, RI). Percent total carbon, organic carbon, total nitrogen, and the carbon/nitrogen ratio were measured via dry combustion and direct measurement of total nutrients using an Elementar analyzer (Elementar, Langenselbold, Germany). DOC was measured using diluted meltwater on a Shimadzu total organic carbon (TOC) analyzer (Shimadzu Corporation, Kyoto, Japan). Electrical conductivity (EC) was measured using a digital benchtop meter with a potentiometric probe submerged in a diluted soil solution (Hanna Instruments, Woonsocket, RI).

### Cell separation from the soil matrix for enumeration via microscopy.

For cell enumeration, cells were separated from the permafrost soil matrix using Nycodenz (Accurate Chemical, Westbury, NY) density cushion centrifugation as described previously (52–56). To separate cells from soil debris, we disrupted 1.5 g of soil in a mild detergent consisting of 2 ml of 0.05% Tween 80 and 50 mM tetrasodium pyrophosphate buffer (TTSP) (53, 57) and sonicated it for 1 min at 20 V using a QSonica ultrasonicator (QSonica, Newtown, CT) with a 0.3-cm-diameter probe. Sonicated samples were centrifuged at 750 × *g* for 7 min at 4°C to remove large particles and debris. We extracted 600 μl of the supernatant and layered it over 600 μl of 1.3 g/liter Nycodenz solution in a 2-ml tube. The tubes were centrifuged at 14,000 × *g* for 30 min at 4°C. We transferred 600 μl of the upper and middle phases containing bacterial cells into a sterile 2-ml tube and centrifuged it at 10,000 × *g* for 15 min at 4°C. The supernatant was discarded, and the pellet was resuspended in 1 ml of 0.85% NaCl solution.

### LIVE/DEAD staining.

A LIVE/DEAD BacLight bacterial viability kit (Invitrogen Detection Technologies, Carlsbad, CA) was used to differentially stain live and dead cells. We added 3 μl of a 1:1 mixture of 3.34 mM SYTO 9 and 20 mM propidium iodide solution to 1-ml cell suspensions according to the manufacturer’s protocol. The stained suspensions were incubated at room temperature for 15 min in the dark to allow the dyes to permeate the cells and bind to DNA.

### DAPI staining.

DAPI staining was performed to obtain total cell counts. After removal of soil debris (as described above [see “Cell Separation from the Soil Matrix for Enumeration via Microscopy”]), we added 3 μl of 14.3 mM DAPI stock solution to each 1 ml of cell suspension. The stained suspensions were incubated in the dark at room temperature for 15 min.

### Cell enumeration.

We diluted and vacuum filtered the stained suspensions onto a 25-mm-diameter 0.2-μm-pore-size black polycarbonate membrane, which we placed on a slide with sterile forceps. Samples were observed at ×1,000 total magnification on a single focal plane using a Zeiss Axio Imager M2 fluorescence microscope coupled to an Apotome 2.0 system with appropriate filters for each stain (Zeiss, Oberkochen, Germany). We counted 15 fields of view for LIVE/DEAD- and DAPI-stained cells for each sample (58, 59). The average number of cells per field of view was multiplied by the area of the filter, and the dilution factor was then corrected for dry weight to calculate the average number of cells per gram (dry weight).

### Cell separation from the soil matrix for propidium monoazide treatment and endospore enrichment.

While Nycodenz density centrifugation is effective at removing soil debris, making it ideal for microscopic visualization, it is biased against endospores and heavily attached cells (60). To separate cells from the soil matrix for downstream propidium monoazide treatment and endospore enrichment experiments, we used a second, less-biased method (61). We disrupted 5 g of sample in 25 ml of 1% sodium hexametaphosphate buffer (SHMP) and sonicated it for 1 min at 20 V using a QSonica ultrasonicator with a 0.6-cm probe. The samples were left for 15 min to allow large particles and debris to settle before transferring the supernatant to a clean 50-ml tube. We added 15 ml of 1% SHMP to the pellet and sonicated it again with a 0.6-cm probe for 1 min at 20 V. The mixture was incubated for another 15 min to allow debris to settle, and then we combined the new supernatant with the supernatant from the previous step. To further remove large particles and debris, we centrifuged the combined supernatant at 20 × *g* for 2 min. We then divided the supernatant equally into two 50-ml tubes as an experimental group (which received either the propidium monoazide treatment or the endospore enrichment) and a control group (which received no treatment). These tubes were centrifuged at 10,000 × *g* for 15 min to pellet the biomass. The biomass pellet was either stored at −20°C to await DNA extraction (in the case of the control pellets) or immediately used for downstream treatments.

### Depletion of DNA from dead cells via propidium monoazide treatment.

To deplete DNA from dead cells, we treated cells extracted using the SHMP method with propidium monoazide (PMAxx; Biotium Inc., Hayward, CA), which is a DNA-intercalating dye similar to the nucleic acid dye propidium iodide (62). It is selectively permeable, passing through the impaired membranes of dead cells, but it is unable to penetrate the membranes of living cells. In the presence of intense bright light, the azide group enables propidium monoazide to covalently cross-link double-stranded DNA (dsDNA), preventing its amplification via PCR (63).

For the propidium monoazide treatment, we resuspended the extracted cell pellets in 500 μl of 0.85% NaCl solution and placed them in clear 1.5-ml microcentrifuge tubes. We added 2.5 μl of 20 mM propidium monoazide solution to each microcentrifuge tube, resulting in a final concentration of 100 μM. We increased the concentration from the commonly used 50 μM due to the presence of leftover soil debris following cell extraction, as recommended for environmental samples by Heise et al. and Bae and Wuertz (33, 64). The tubes were incubated in the dark at room temperature for 10 min. After incubation, we placed the tubes on a sheet of foil in an ice bucket to prevent warming. A 500-W halogen work lamp was placed 20 cm above the samples for 15 min. Every 5 min, we mixed the samples gently to ensure even light distribution. Following light exposure, we centrifuged samples at 10,000 × *g* for 15 min and discarded the supernatant. We stored these propidium monoazide-treated pellets at −20°C until they were used in downstream DNA extractions.

Cell extraction from soil fails to remove all soil particles, which can subsequently block light penetration and prevent propidium monoazide from cross-linking to DNA. To verify that our propidium monoazide treatment was effective in the presence of the small number of remaining particles, we extracted cells from temperate control soils collected from the CSUN campus, spiked the sample with 3.6 × 10^8^ isopropanol-killed E. coli cells, and treated the mixture with propidium monoazide. We also performed the treatment without E. coli spike-ins on the same temperate control samples.

To prepare isopropanol-killed E. coli cells, overnight cultures were centrifuged at 5,000 × *g* for 10 min to pellet the cells. Following centrifugation, the supernatant was removed, the pellet was resuspended in 10 ml of isopropanol, and the cells were incubated for 10 min. Isopropanol-killed E. coli cells were pelleted again by centrifugation at 5,000 × *g* for 10 min. The supernatant was removed, and the resulting pellet was resuspended in 0.85% NaCl to wash off residual isopropanol. The isopropanol-killed cells were centrifuged once more at 5,000 × *g* for 10 min, the supernatant was removed, and the pellet was resuspended in 1 ml 0.85% NaCl.

The amount of DNA removed was determined by comparing the number of copies of the 16S rRNA gene in the spiked and nonspiked samples before and after treatment (as determined by qPCR [*R*^2^ = 0.99; 61.8% efficiency]—see below for detailed protocols). The number of 16S rRNA gene copies decreased by ∼66% after treatment (Student's *t* test = 3.6; *P* < 0.01), showing that, even in the presence of soil particles, treatment removed 66% of added exogenous DNA.

### Endospore enrichment.

To separate endospores from vegetative cells, we used a treatment involving three steps: physical, enzymatic, and chemical cell lysis based on the protocol of Wunderlin et al. (61). The first physical treatment uses heat to lyse vegetative cells. Next, lysozyme dissolves the cell membrane, followed by a solution of sodium hydroxide (NaOH) and sodium dodecyl sulfate (SDS) to further disrupt cellular membranes. Finally, a DNase treatment is used to degrade the DNA from the ruptured cells.

We resuspended cell pellets extracted using the SHMP method with 900 μl of 1× Tris-EDTA buffer (10 mM Tris and 1 mM EDTA, pH 8) and placed them into 2-ml tubes. The tubes were placed in a heat block at 60°C for 10 min with shaking at 80 rpm. After incubation, we let the tubes cool for 15 min to 37°C before adding 100 μl of lysozyme solution (20 mg/ml in 1× Tris-EDTA [TE] buffer) and incubating them in a heat block at 37°C for 60 min with shaking at 80 rpm. After lysis, 250 μl of 3 N NaOH and 100 μl of 10% SDS were added to the sample, reaching a final volume of 1.35 ml, which we incubated at room temperature for 60 min at 80 rpm. After the final incubation, we centrifuged the solution at 10,000 × *g* for 15 min to pellet cell debris and discarded the supernatant. We resuspended the pellet with 450 μl of water, 50 μl of 1× DNase reaction buffer, and 1 μl DNase enzyme (New England Biolabs, Ipswich, MA) and left it for 15 min to remove DNA from lysed and dead cells. Following the DNase treatment, we centrifuged the tubes for 15 min at 10,000 × *g* to pellet endospores and discarded the supernatant. The pellet was then resuspended in 1 ml 0.85% NaCl solution to wash away residual DNase enzyme. We centrifuged the suspension at 10,000 × *g* for 15 min, discarded the supernatant, and stored the endospore-enriched pellet at −20°C in a sterile Eppendorf tube until it was used for downstream DNA extraction.

To confirm that endospore enrichment depletes vegetative cells, we extracted cells from temperate control soils collected on the CSUN campus, spiked the samples with 1.4 × 10^7^ live E. coli cells, and performed the endospore enrichment. We used the same treatment on the temperate control soils without E. coli spike-ins. By comparing the number of copies of 16S rRNA genes before and after treatment in the spiked and nonspiked samples, we were able to measure the amount of DNA removed (as determined by qPCR [*R*^2^=0.99; 54.5% efficiency]—see below for detailed protocols). Endospore enrichment treatment resulted in significant (82%) removal of DNA from vegetative E. coli cells (Student's *t* test; *P* < 0.01), demonstrating the effectiveness of endospore enrichment in removing vegetative cells.

### DNA extraction.

We performed DNA extractions using a modified bead-beating protocol capable of lysing endospores, cysts, and cells with thickened walls, all of which are known to exist in permafrost (8, 15, 40, 65). We resuspended propidium monoazide-treated, endospore-enriched, and control pellets in 775 μl of lysis buffer (0.75 M sucrose, 20 mM EDTA, 40 mM NaCl, 50 mM Tris) and transferred them to an MP Bio Lysis Matrix E tube. We added 100 μl of 20-mg/ml lysozyme and incubated the samples at 37°C for 30 min. Following incubation, 100 μl 10% SDS was added, and the samples were homogenized in an MP Biomedicals FastPrep 24 homogenizer for 20 s at 4.0 m/s. We placed the samples in a heat block at 99°C for 2 min and allowed them to cool at room temperature for 5 min. We added 25 μl of 20-mg/ml proteinase K (final concentration, 0.5 mg/ml) and incubated samples at 55°C overnight. The next day, we centrifuged the tubes at 10,000 × *g* for 15 min and transferred the supernatant to a clean 2-ml Eppendorf tube. We used a FastDNA spin kit for soil (MP Biomedicals, Santa Ana, CA) for DNA extractions from the lysed cells, following the manufacturer’s protocols but omitting the initial lysis step. The DNA was cleaned using a Power Clean DNA cleanup (Mo Bio, Carlsbad, CA) and quantified using a Qubit 2.0 fluorometer (Thermo Fisher Scientific, Canoga Park, CA).

### PCR amplification and sequencing of the 16S rRNA gene.

Variable region four (V4) of bacterial and archaeal 16S rRNA genes from the propidium monoazide-treated, endospore-enriched, and control groups was amplified as described by Caporaso et al. (66) but with the addition of 2 μl of 20-mg/ml bovine serum albumin in each PCR mixture. We used the golay barcoded primer set 515F/806R (515F, GTGYCAGCMGCCGCGGTAA; 806R, TAATCTWTGGGVHCATCAGG) (see Table S1 in the supplemental material) with an added degeneracy to enhance amplification of archaeal sequences on the 515F primer and thermal-cycling steps recommended by the Earth Microbiome Project protocol version 4.13 (66). The amplified PCR products from triplicate reactions for each sample were pooled at approximately equal concentrations, as measured using a PicoGreen dsDNA quantification assay kit (Thermo Fisher Scientific, Canoga Park, CA). We quantified the pooled 16S rRNA gene amplicons by qPCR using an Illumina library quantification kit (Kapa Biosystems, Wilmington, MA) on a CFX96 real-time PCR detection system (Bio-Rad, Hercules, CA). We performed paired-end sequencing (2 × 150 bp) of 16S rRNA gene amplicons using a 300-bp v2 reagent kit on an Illumina MiSeq instrument.

We demultiplexed and quality filtered raw fastq data using the Quantitative Insights into Microbial Ecology (QIIME) software package version 1.9.1 (67). Sequences were truncated at the first position with a quality score of less than 3. Then, forward and reverse sequences were merged with a minimum merged sequence length of 200 bp, a minimum overlapping length of 20 bp, and a maximum of one difference in the aligned sequence. All sequences that passed quality filtering were *de novo* clustered into operational taxonomic units (OTUs) at 97% sequence identity using USEARCH (68). We assigned taxonomy using the RDP classifier (69) with a confidence score of 0.5 (70, 71). For phylogenetic metrics of diversity, a phylogenetic tree was constructed using FastTree (72) as implemented in QIIME. We rarefied samples to equal depths (*n* = 5,000 sequences/sample) for all subsequent analyses. One OTU was abundant (>3% relative abundance) only in the blank samples and samples that had the lowest DNA yields (see Fig. S1 in the supplemental material). This OTU, from the genus *Burkholderia*, was removed from all other samples because it was likely a result of laboratory contamination (73).

### Quantitative PCR of the 16S rRNA gene.

qPCR of the V4 region of the 16S rRNA gene was conducted on propidium monoazide-treated and endospore-enriched temperate control soil DNA after receiving a spike-in of E. coli to evaluate our methods. Amplification was performed using the 515F/806R primer set. Triplicate qPCRs were done in 25-μl volumes (12.5 μl of GoTaq qPCR master mix [Promega, Madison, WI], 1.25 μl of each primer [19, 66], 5 μl nuclease-free water, and 5 μl of template DNA [≥0.7 ng]) in 96-well plates on a CFX96 real-time PCR detection system (Bio-Rad, Hercules, CA). The thermal cycler program was as follows: 95°C for 2 min, 40 cycles of 95°C for 15 s and 60°C for 60 s, and a melting-curve analysis (60 to 95°C). Quantified full-length E. coli 16S rRNA gene amplicons were used to make a standard curve. A negative control lacking template DNA was performed along with each qPCR.

### Statistical analysis.

We tested for differences in soil chemical characteristics between age categories with a Kruskal-Wallis test and Dunn’s *post hoc* test for nonparametric data using the PMCMR package in R (74). *P* values were corrected using the FDR. Significant differences in the ratio of live to dead cells, the proportion of live cells, and the direct cell counts for each stain between age categories was also tested using the same methods.

We used the Shannon diversity index and phylogenetic-diversity (PD) metrics to calculate alpha diversity using the alpha_diversity.py script in QIIME (75). Differences in alpha diversity between age categories and treatments were tested by the same methods as for soil chemistry. We computed beta diversity using the weighted UniFrac metric (76). Differences between samples were visualized by principal-coordinate analysis (PCoA) using the phyloseq package in R (77). We also used nonmetric multidimensional scaling coordinates of microbial community composition between samples for PERMANOVA as a function of soil chemistry data using the function adonis from the vegan package in R (76, 78).

Differences in the relative abundances of specific taxa between treatments and age categories were tested using a linear mixed effect model on rank-transformed taxon abundances and nested treatment as a factor within age using the nlme package in R (79). *P* values were corrected using the FDR. The specific taxa indicated were tested for various pairwise comparisons between treatments and untreated controls for each age category with a Mann-Whitney-Wilcoxon test using the wilcoxon.test function in R. Differences in 16S rRNA gene copy numbers, as quantified using qPCR, between propidium monoazide-treated samples and untreated controls (including samples given a spike-in with dead E. coli cells) and the endospore-enriched samples (given a spike-in with live E. coli cells) were tested with a *t* test, using the t.test function in R.

### Accession number(s).

16S rRNA gene sequence data were uploaded to the NCBI Sequence Read Archive under accession number SRP158034.

## Supplementary Material

Supplemental file 1
