# Supplementary material for: Changes in the Active, Dead, and Dormant Microbial Community Structure across a Pleistocene Permafrost Chronosequence
Source: Appl Environ Microbiol. 2019 Mar 22;85(7):e02646-18. doi: 10.1128/AEM.02646-18 (PMC6585489; doi:10.1128/AEM.02646-18)
Supplement: Supplemental file 1 [file AEM.02646-18-s0001.pdf]

Supplementary Material

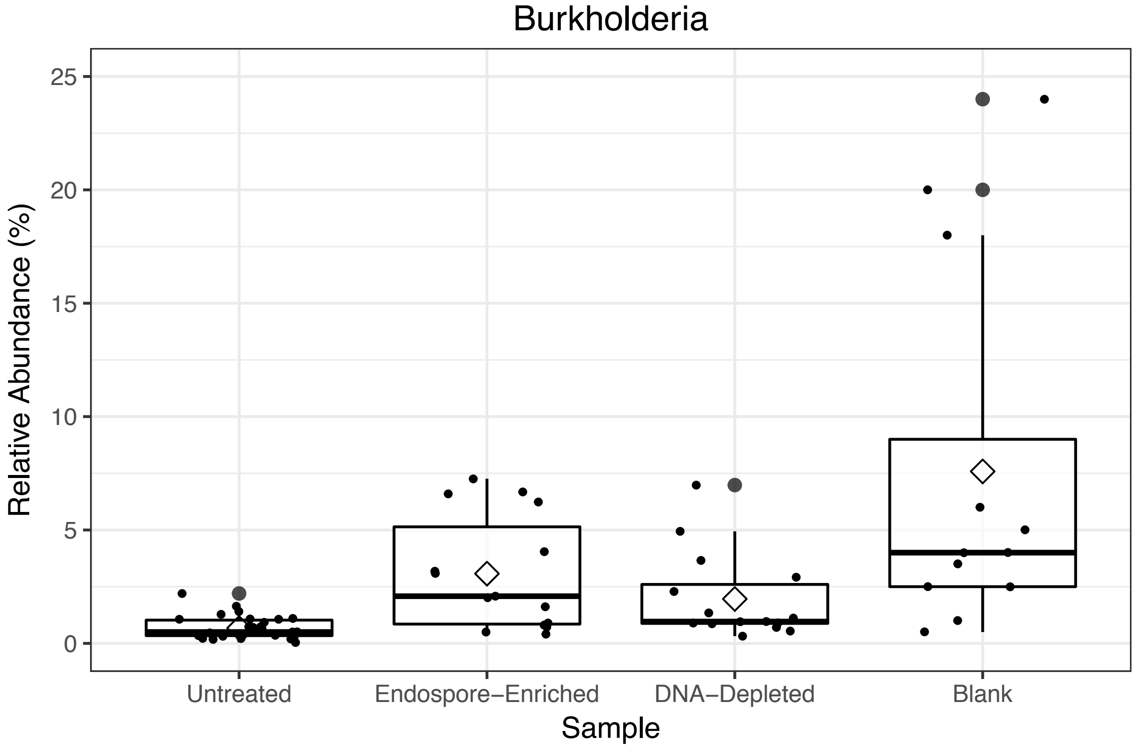

Figure S1. Relative abundance of the genera Burkholderia based on 16S rRNA gene sequences. This OTU was only abundant in samples with very low DNA yields (blank controls as well as endospore-enriched and DNA-depleted samples from the oldest time point) indicating it was likely the result of contamination. Therefore, this OTU was removed from subsequent analyses.

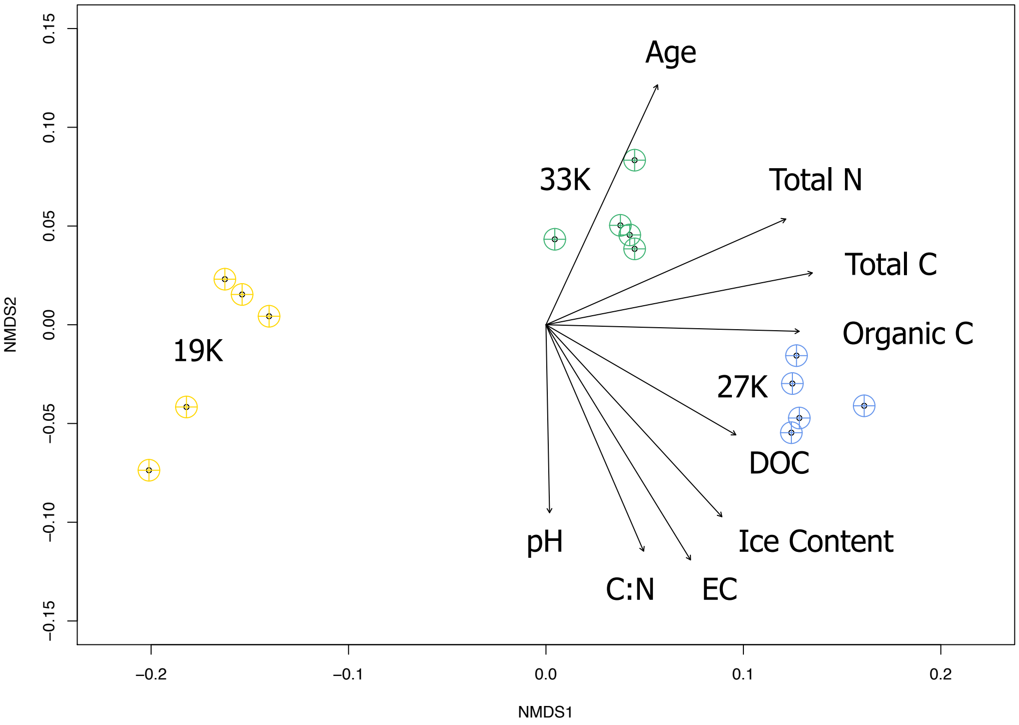

Figure S2. Non-metric multidimensional scaling (NMDS) based on weighted Unifrac values of 16S rRNA gene sequences.

Table S1. Table of barcodes used on the 806R primer for each sample during multiplexed sequencing.

SampleID	BarcodeSequence	LinkerPrimerSequence	Treatment	Age
D.19K6	TGTGTAGCCATG	GGACTACHVGGGTWTCTAAT	Endospore-Enriched	19K
D.19K7	GAGCATTACATG	GGACTACHVGGGTWTCTAAT	Endospore-Enriched	19K
D.19K8	CGGAGAGACATG	GGACTACHVGGGTWTCTAAT	Endospore-Enriched	19K
D.19K9	AGTCATCGAATG	GGACTACHVGGGTWTCTAAT	Endospore-Enriched	19K
D.19K10	CAGCAGTCTTCG	GGACTACHVGGGTWTCTAAT	Endospore-Enriched	19K
D.27K6	AGTGTCGATTCG	GGACTACHVGGGTWTCTAAT	Endospore-Enriched	27K
D.27K7	TGTCAGCTGTCG	GGACTACHVGGGTWTCTAAT	Endospore-Enriched	27K
D.27K8	AATAGCATGTCG	GGACTACHVGGGTWTCTAAT	Endospore-Enriched	27K
D.27K9	AACACTCGATCG	GGACTACHVGGGTWTCTAAT	Endospore-Enriched	27K
D.27K10	TCTGGTGCATCG	GGACTACHVGGGTWTCTAAT	Endospore-Enriched	27K
D.33K6	TTGACGACATCG	GGACTACHVGGGTWTCTAAT	Endospore-Enriched	33K
D.33K7	ATAACATGTGCG	GGACTACHVGGGTWTCTAAT	Endospore-Enriched	33K
D.33K8	ATAGACACTCCG	GGACTACHVGGGTWTCTAAT	Endospore-Enriched	33K
D.33K9	TGTACATCGCCG	GGACTACHVGGGTWTCTAAT	Endospore-Enriched	33K
D.33K10	AGTAGACTTACG	GGACTACHVGGGTWTCTAAT	Endospore-Enriched	33K
D.Blank	GATAACTGTACG	GGACTACHVGGGTWTCTAAT	Endospore-Enriched	Blank
DC.19K6	GACATTGTCACG	GGACTACHVGGGTWTCTAAT	NonEnriched-Control	19K
DC.19K7	GGAGAGATCACG	GGACTACHVGGGTWTCTAAT	NonEnriched-Control	19K
DC.19K8	TGATAATGCACG	GGACTACHVGGGTWTCTAAT	NonEnriched-Control	19K
DC.19K9	CTGATGTACACG	GGACTACHVGGGTWTCTAAT	NonEnriched-Control	19K
DC.19K10	CACCTGTAGTAG	GGACTACHVGGGTWTCTAAT	NonEnriched-Control	19K
DC.27K6	CGATATCAGTAG	GGACTACHVGGGTWTCTAAT	NonEnriched-Control	27K
DC.27K7	AGTGCGTTCTAG	GGACTACHVGGGTWTCTAAT	NonEnriched-Control	27K
DC.27K8	AAGAGTCTCTAG	GGACTACHVGGGTWTCTAAT	NonEnriched-Control	27K
DC.27K9	CTGCATACTGAG	GGACTACHVGGGTWTCTAAT	NonEnriched-Control	27K
DC.27K10	ACTGTACATGAG	GGACTACHVGGGTWTCTAAT	NonEnriched-Control	27K
DC.33K6	TCTCATGTGGAG	GGACTACHVGGGTWTCTAAT	NonEnriched-Control	33K
DC.33K7	AGACAGTAGGAG	GGACTACHVGGGTWTCTAAT	NonEnriched-Control	33K
DC.33K8	CTGTCGTGTCAG	GGACTACHVGGGTWTCTAAT	NonEnriched-Control	33K
DC.33K9	CGCGTATCTCAG	GGACTACHVGGGTWTCTAAT	NonEnriched-Control	33K
DC.33K10	GACTCTGCTCAG	GGACTACHVGGGTWTCTAAT	NonEnriched-Control	33K
DC.Blank	TAAGTACTGCAG	GGACTACHVGGGTWTCTAAT	NonEnriched-Control	Blank
V.19K6	TCACAGACAATG	GGACTACHVGGGTWTCTAAT	DNA-Depleted	19K
V.19K7	ACGATCATCTGG	GGACTACHVGGGTWTCTAAT	DNA-Depleted	19K
V.19K8	TGCATACACTGG	GGACTACHVGGGTWTCTAAT	DNA-Depleted	19K
V.19K9	AGCTATGTATGG	GGACTACHVGGGTWTCTAAT	DNA-Depleted	19K

V.19K10	ACACATAAGTCG	GGACTACHVGGGTWTCTAAT	DNA-Depleted	19K
V.27K6	GCGTAACTCTCG	GGACTACHVGGGTWTCTAAT	DNA-Depleted	27K
V.27K7	ATGTGCTGCTCG	GGACTACHVGGGTWTCTAAT	DNA-Depleted	27K
V.27K8	TACTGAGCCTCG	GGACTACHVGGGTWTCTAAT	DNA-Depleted	27K
V.27K9	TTCTAGAGTGCG	GGACTACHVGGGTWTCTAAT	DNA-Depleted	27K
V.27K10	AGTCTGTCTGCG	GGACTACHVGGGTWTCTAAT	DNA-Depleted	27K
V.33K6	TATACCGCTGCG	GGACTACHVGGGTWTCTAAT	DNA-Depleted	33K
V.33K7	GATCCTCATGCG	GGACTACHVGGGTWTCTAAT	DNA-Depleted	33K
V.33K8	CAGTCTAGTACG	GGACTACHVGGGTWTCTAAT	DNA-Depleted	33K
V.33K9	GCACGTTCTACG	GGACTACHVGGGTWTCTAAT	DNA-Depleted	33K
V.33K10	TTCGCAGATACG	GGACTACHVGGGTWTCTAAT	DNA-Depleted	33K
V.Blank	ATACAGCATACG	GGACTACHVGGGTWTCTAAT	DNA-Depleted	Blank
VC.19K6	CCTGATCACACG	GGACTACHVGGGTWTCTAAT	NonDepleted-Control	19K
VC.19K7	GCTCTAGTAACG	GGACTACHVGGGTWTCTAAT	NonDepleted-Control	19K
VC.19K8	GTGTACATAACG	GGACTACHVGGGTWTCTAAT	NonDepleted-Control	19K
VC.19K9	TCTATGCGAACG	GGACTACHVGGGTWTCTAAT	NonDepleted-Control	19K
VC.19K10	GATGTTCGCTAG	GGACTACHVGGGTWTCTAAT	NonDepleted-Control	19K
VC.27K6	CTAGTGACCTAG	GGACTACHVGGGTWTCTAAT	NonDepleted-Control	27K
VC.27K7	TCAGCTGACTAG	GGACTACHVGGGTWTCTAAT	NonDepleted-Control	27K
VC.27K8	ACACGACTATAG	GGACTACHVGGGTWTCTAAT	NonDepleted-Control	27K
VC.27K9	CGTACTCTCGAG	GGACTACHVGGGTWTCTAAT	NonDepleted-Control	27K
VC.27K10	GATCTAATCGAG	GGACTACHVGGGTWTCTAAT	NonDepleted-Control	27K
VC.33K6	TATATGTGCGAG	GGACTACHVGGGTWTCTAAT	NonDepleted-Control	33K
VC.33K7	CTACTCCACGAG	GGACTACHVGGGTWTCTAAT	NonDepleted-Control	33K
VC.33K8	TCTTCGCAGCAG	GGACTACHVGGGTWTCTAAT	NonDepleted-Control	33K
VC.33K9	CTATGAGTCCAG	GGACTACHVGGGTWTCTAAT	NonDepleted-Control	33K
VC.33K10	GTCATGCTCCAG	GGACTACHVGGGTWTCTAAT	NonDepleted-Control	33K
VC.Blank	TCATACAGCCAG	GGACTACHVGGGTWTCTAAT	NonDepleted-Control	Blank

Table S2. Average percent difference in relative abundance between the endospore-enriched samples and non-enriched controls across the three age categories. A negative value shows underrepresentation in the endospore-enriched sample compared to the non-enriched control while a positive value shows overrepresentation. Values are averages of five replicate cores. U-value is a test statistic generated during a Mann-Whitney-Wilcoxon test which represents the level of difference between two groups. Lower U-values indicate that two groups are more different.

Taxa	19K (%)	U-value	27K (%)	U-value	33K (%)	U-value
Proteobacteria	-4.3 ± 2.2	6	-4.0 ± 0.3 **	0	-6.2 ± 2.1	5
Alphaproteobacteria	3.6 ± 0.7	6	-3.8 ± 0.3 **	0	-0.6 ± 0.3	9
Deltaproteobacteria	-8.8 ± 0.3 **	0	-0.2 ± 0.1 *	1.5	-6.0 ± 0.7 **	0
Bacteroidetes	-3.9 ± 0.7 **	0	0.0 ± 0.0	4.5	-2.9 ± 0.9 **	0
Acidobacteria	-3.2 ± 0.3 *	0	-0.1 ± 0.0 *	1.5	-0.1 ± 0.1	4.5
Chloroflexi	-1.4 ± 0.3 **	0	-0.6 ± 0.1 **	0	-0.1 ± 0.1	7
Planctomycetes	-1.3 ± 0.1 **	0	-0.8 ± 0.1 *	0	-0.3 ± 0.1	4

(Mann-Whitney-Wilcoxon test, ** = $p < 0.01$, * = $p < 0.05$)
